# Comparative Effectiveness of the Proximal Femoral Nail and Dynamic Hip Screw Fixation in Intertrochanteric Femur Fractures: A Systematic Review and Meta-Analysis

**DOI:** 10.7759/cureus.94767

**Published:** 2025-10-17

**Authors:** Shahmeen Rasul, Shashwat Shetty, Mohamad Mortada, Ahmad A Quzli, Siddhesh V Kulkarni, Osasenaga Bencharles, Hassan Jouni, Farhan Saleem

**Affiliations:** 1 Trauma and Orthopaedics, University Hospitals of Derby and Burton NHS Foundation Trust, Burton-on-Trent, GBR; 2 Orthopaedics, Hillingdon Hospitals NHS Foundation Trust, Uxbridge, GBR; 3 Vascular Surgery, Nottingham University Hospitals NHS Trust, Nottingham, GBR; 4 Trauma and Orthopaedics, Wirral University Teaching Hospital NHS Foundation Trust, Wirral, GBR; 5 Trauma and Orthopaedics, Hillingdon Hospitals NHS Foundation Trust, Uxbridge, GBR; 6 Medicine and Surgery, University Hospitals of Derby and Burton NHS Foundation Trust, Burton-on-Trent, GBR; 7 General Surgery, Queen's Hospital Burton, Burton, GBR; 8 Orthopaedic Surgery, Lahore General Hospital, Lahore, PAK

**Keywords:** dynamic hip screw, intertrochanteric fractures, meta-analysis, orthopedic surgery, proximal femoral nail

## Abstract

This systematic review and meta-analysis compared the clinical effectiveness of proximal femoral nail (PFN) versus dynamic hip screw (DHS) fixation in patients with intertrochanteric femur fractures. A comprehensive literature search was conducted across multiple databases from January 2010 to September 2025, identifying studies that directly compared PFN and DHS fixation outcomes. Thirty-three studies met the inclusion criteria, comprising randomized controlled trials, prospective cohorts, and retrospective comparative studies from diverse geographic regions.

The pooled analysis demonstrated several significant advantages favoring PFN fixation. Operative time was significantly shorter with PFN compared to DHS (mean difference (MD): -12.30 minutes, 95% confidence interval (CI): -17.33 to -7.28), while intraoperative blood loss was substantially lower (MD: -115.01 mL, 95% CI: -132.05 to -97.98). Patients treated with PFN achieved full weight-bearing significantly earlier than those receiving DHS.

Safety outcomes showed PFN was associated with significantly lower total complication rates (risk ratio (RR): 0.46, 95% CI: 0.31-0.68) and reduced infection risk. However, no significant differences were observed between groups regarding implant failure rates, mortality, or long-term functional outcomes, as measured by the Harris Hip Score. High heterogeneity was noted across most outcomes, reflecting variations in study populations and methodologies.

These findings suggest that PFN offers superior perioperative outcomes and early recovery advantages compared to DHS, while maintaining comparable long-term functional results and survival rates in patients with intertrochanteric femur fractures.

## Introduction and background

Intertrochanteric femur fractures represent one of the most challenging orthopedic injuries in the elderly population, with an estimated annual incidence exceeding 1.6 million cases globally [1]. These fractures, anatomically defined as occurring between the greater and lesser trochanters of the proximal femur, constitute approximately 50% of all hip fractures, and disproportionately affect individuals over 65 years of age [2-3]. The demographic shift toward an aging population has resulted in a projected doubling of intertrochanteric fracture incidence by 2050, creating substantial healthcare and socioeconomic burdens worldwide [4].

The pathophysiology of intertrochanteric fractures in elderly patients is multifactorial, typically resulting from low-energy trauma in the setting of osteoporotic bone. Age-related bone density reduction, particularly in Ward's triangle and the greater trochanteric region, creates biomechanical weakness, predisposing to fracture with minimal force [5]. Additionally, sarcopenia, visual impairment, and medication-related fall risk further compound fracture susceptibility in this vulnerable population. The clinical consequences extend beyond the immediate injury, with reported one-year mortality rates ranging from 14% to 36%, and significant functional decline in survivors [6-7].

Surgical management remains the definitive treatment for intertrochanteric fractures, aimed at achieving stable fixation to facilitate early mobilization and prevent prolonged immobilization-related complications. The two predominant fixation strategies are extramedullary devices, exemplified by the dynamic hip screw (DHS), and intramedullary implants, primarily represented by the proximal femoral nail (PFN) [8]. The selection between these approaches continues to generate considerable debate within the orthopedic community. PFN insertion requires closed reduction techniques and fluoroscopic guidance, with entry point selection (piriformis fossa versus greater trochanter tip) influencing fracture reduction quality and implant positioning. The procedure demands precise guidewire placement in the center-center or inferior-center position on anteroposterior and lateral radiographs, to minimize cut-out complications [8]. DHS implantation necessitates open reduction through a lateral approach, with direct fracture visualization, allowing anatomical reduction but requiring more extensive soft tissue dissection [9].

The DHS system, established as the traditional gold standard since the 1970s, employs a large-diameter cancellous screw inserted into the femoral head through a side plate, secured to the lateral femoral cortex [9]. This design facilitates controlled collapse and compression at the fracture site through its sliding mechanism, promoting bone healing via dynamic compression principles. The DHS demonstrates excellent outcomes for stable fracture patterns (AO/OTA 31-A1), with an intact posteromedial cortex, and has accumulated extensive long-term follow-up data supporting its efficacy [10-11].

Conversely, the PFN represents an evolution in intramedullary fixation technology, introduced to address perceived limitations of extramedullary devices. The PFN utilizes a cephalomedullary nail inserted through the piriformis fossa or tip of the greater trochanter, with proximal interlocking achieved through either a single lag screw or a dual screw/helical blade configuration [12]. This intramedullary approach theoretically offers biomechanical advantages, including a reduced lever arm, enhanced load-sharing characteristics due to proximity to the femoral mechanical axis, and preservation of the fracture hematoma through minimally invasive techniques [13].

The biomechanical rationale favoring PFN centers on load distribution principles. Finite element analyses demonstrate that intramedullary devices experience significantly lower bending moments and stress concentrations compared to extramedullary implants, particularly in unstable fracture patterns with lateral wall compromise [14]. In addition, the relatively shorter surgical exposure required for PFN implantation may lower operative morbidity, particularly in elderly patients with limited physiological reserves. At present, PFN systems are widely applied in clinical practice and are available from multiple manufacturers, offering variations in length, diameter, neck-shaft angle, number of cephalic screws, rotational stability features, and construction materials [15]. Although PFN is considered to have certain theoretical advantages over DHS, debate continues in the literature - especially based on clinical trial evidence - regarding whether PFN truly offers superior outcomes. Data from recent large registry analyses, such as the study by Grønhaug et al., indicate that PFN is primarily recommended for unstable trochanteric and subtrochanteric fractures, while it may not be advantageous for stable fractures or specific subtypes [16].

The objective of this study was to perform an updated meta-analysis, comparing the effectiveness of PFN (including proximal femoral nail antirotation (PFNA)) versus DHS in the management of intertrochanteric femur fractures.

## Review

Methodology

Study Design

This systematic review and meta-analysis was conducted in accordance with the Preferred Reporting Items for Systematic Reviews and Meta-Analyses (PRISMA) guidelines [17]. The protocol of this meta-analysis was registered with PROSPERO (CRD420251165657).

Search Strategy

A comprehensive literature search was performed across multiple electronic databases, including PubMed/MEDLINE, Embase, Cochrane Central Register of Controlled Trials (CENTRAL), Web of Science, and Scopus, from January 1, 2010, to September 10, 2025. The search strategy combined Medical Subject Headings (MeSH) terms and free-text keywords related to intertrochanteric femur fractures, PFNs, and DHS. The search terms included: "intertrochanteric fracture*", "proximal femoral nail*", "PFN", "PFNA", "dynamic hip screw*", "DHS", "sliding hip screw", and "extramedullary fixation." Reference lists of included studies and relevant systematic reviews were manually screened for additional eligible studies. No language restrictions were applied, and studies published in languages other than English were translated when necessary. The search was performed by two authors independently. Any disagreement between the two authors was resolved through discussion.

Eligibility Criteria

Two independent reviewers screened all titles and abstracts identified through the database searches, using predetermined inclusion and exclusion criteria, as shown in Table 1. Full-text articles of potentially eligible studies were obtained and independently assessed for inclusion by the same reviewers. Disagreements were resolved through discussion, and when consensus could not be reached, a third reviewer was consulted.

**Table 1:** Eligibility criteria

Criteria Type	Criterion	Description
Inclusion	1	Patients with radiologically confirmed intertrochanteric femur fractures
Inclusion	2	Comparative studies directly comparing proximal femoral nail (PFN) systems with dynamic hip screw (DHS) fixation
Inclusion	3	Reported clinical outcomes including at least one of the following: operative parameters, functional outcomes, complications, or mortality
Inclusion	4	Study designs including randomized controlled trials, quasi-randomized trials, or prospective comparative cohort studies
Exclusion	1	Studies that compared different types of intramedullary nails without a DHS comparison
Exclusion	2	Case series, case reports, or retrospective studies without appropriate control groups
Exclusion	3	Studies with insufficient data for meta-analysis despite contact with authors
Exclusion	4	Duplicate publications reporting on the same patient population

Data Extraction

Data extraction was performed independently by two reviewers, using a standardized data extraction form developed specifically for this review. The following information was extracted from each included study: study characteristics (author, year of publication, country, study design, and sample size), patient demographics (age, gender, and fracture classification), intervention details (type of PFN system and DHS specifications), operative parameters (operative time, blood loss, and fluoroscopy time), functional outcomes (Harris Hip Score and mobility scores), complications (infection, cut-out, implant failure, and reoperation rates), and mortality data. When studies reported outcomes at multiple time points, data from the longest available follow-up were extracted for the primary analysis.

Quality Assessment

The methodological quality of randomized controlled trials (RCTs) was assessed using the Cochrane Risk of Bias tool (RoB 2.0) [18], evaluating bias arising from the randomization process, deviations from intended interventions, missing outcome data, measurement of outcomes, and selection of reported results. For non-randomized comparative studies, the Newcastle-Ottawa Scale was used to assess study quality across three domains: selection of study groups, comparability of groups, and ascertainment of exposure or outcome [19]. Studies were classified as having low, moderate, or high risk of bias, based on predetermined criteria.

Data Analysis

Analysis was performed using RevMan Version 5.4.1 (The Cochrane Collaboration, Oxford, UK). Continuous variables were expressed as mean differences (MDs) with 95% confidence intervals (CIs), while dichotomous outcomes were reported as risk ratios (RRs) with 95% CI. Statistical heterogeneity was assessed using the I² statistic, with values of 25%, 50%, and 75% representing low, moderate, and high heterogeneity, respectively. A random-effects model was employed for all analyses, due to anticipated clinical and methodological heterogeneity between studies.

Results

**Figure 1:**
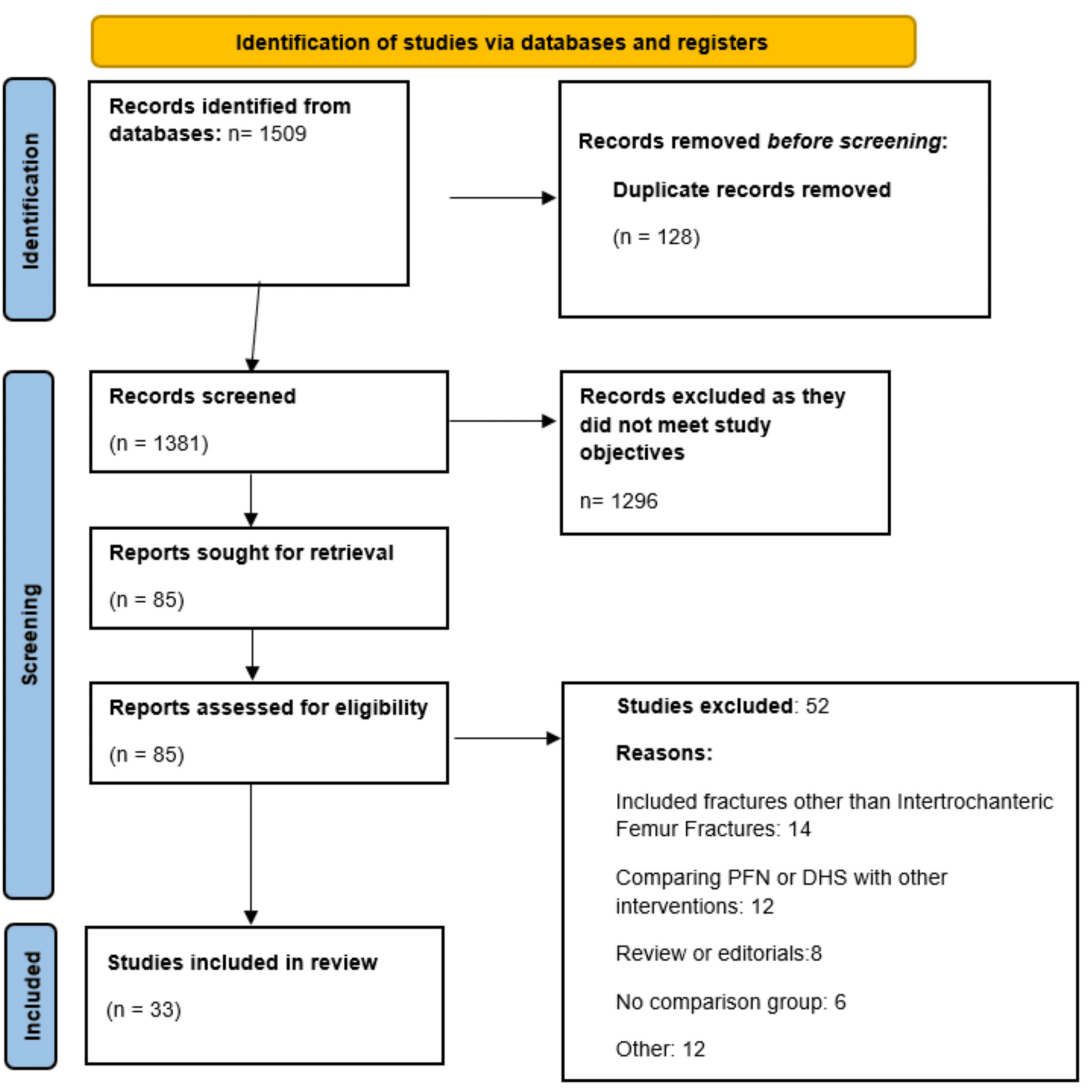
PRISMA flowchart PFN: proximal femoral nail; DHS: dynamic hip screw

From the database searching, 1509 studies were included. After removing 128 duplicate records, the remaining articles were screened using their titles and abstracts. The full text of 85 studies was thoroughly screened. Finally, 33 studies met the inclusion criteria and were included in this meta-analysis. Figure 1 shows the detailed study selection process.

The baseline characteristics of each included study are presented in Table 2. The included studies comprised a mix of RCTs, prospective cohorts, and retrospective cohort studies conducted across multiple regions, predominantly in Asia (India, China, Korea, Singapore, and Iran), as well as Europe (Italy, Turkey, and the United Kingdom), and South America (Brazil). The follow-up durations varied widely, ranging from 3 months to over 56 months, with most studies reporting outcomes at 6 to 24 months. Sample sizes also showed considerable variation, from as few as 12 patients per group to large multicenter trials with up to 300 participants in each arm. The mean age of patients generally reflected an elderly population (mostly between 65 and 85 years), though some studies included younger cohorts, with variability in male-to-female distribution. Both DHS and PFN were evaluated, with many studies including mixed populations of stable and unstable fractures, while others focused specifically on either stable or unstable intertrochanteric fractures. Table 3 presents the quality assessment of the included observational studies.

**Table 2:** Studies characteristics RCT: randomized controlled trial; NR: not reported; PFN: proximal femoral nail; DHS: dynamic hip screw

Author Name	Study Design	Region	Follow-Up	Groups	Sample Size	Mean Age	Male	Stability Classification
Anshul et al. (2022) [20]	Retrospective cohort	India	12 Months	DHS	40	NR	21	Mixed (stable and unstable)
				PFN	40		22	
Canbeyli et al. (2021) [21]	Retrospective cohort	Turkey	12 Months	PFN	58	82.21	NR	Mixed (stable and unstable)
				DHS	49	80.78		
Carulli et al. (2017) [22]	RCT	Italy	3 Months	PFN	71	81.62	29	Mixed (stable and unstable)
				DHS	69	83.41	25	
Chen et al. (2018) [23]	RCT	China	6 Months	PFN	18	63.2	10	Mixed (stable and unstable)
				DHS	18	64.3	9	
Cho and Lee (2016) [24]	Retrospective cohort	Korea	12 Months	PFN	81	81	43	Stable
				DHS	113	84.2	26	
Chua et al. (2013) [25]	Retrospective cohort	Singapore	12 Months	PFN	25	75	13	Unstable
				DHS	38	77	18	
Das et al. (2020) [26]	RCT	India	24 Months	PFN	75	68.6	21	Unstable
				DHS	75	68.8	27	
Duan et al. (2017) [27]	Prospective cohort	China	18 Months	PFN	46	NR	NR	Unstable
				DHS	62			
Duymus et al. (2019) [28]	Retrospective cohort	Turkey	12 Months	PFN	32	71.66	13	Unstable
				DHS	30	72.13	16	
Garg et al. (2022) [29]	Prospective cohort	India	12 Months	PFN	35	70.38	11	Unstable
				DHS	35	69.8	10	
Guerra et al. (2014) [30]	RCT	Brazil	12 Months	PFN	12	NR	1	Mixed (stable and unstable)
				DHS	19		5	
Guo et al. (2017) [31]	Retrospective cohort	China	56 Months	PFN	82	NR	NR	Unstable
				DHS	71			
Huang et al. (2017) [32]	RCT	China	24 Months	PFN	30	75.07	15	Unstable
				DHS	30	74.01	17	
Jonnes et al. (2016) [33]	Prospective cohort	India	12 Months	PFN	15	NR	NR	Mixed (stable and unstable)
				DHS	15			
Kumar et al. (2012) [34]	RCT	India	6 Months	PFN	25	NR	NR	Mixed (stable and unstable)
				DHS	25			
Li et al. (2018) [35]	RCT	China	18 Months	PFN	40	NR	NR	Mixed (stable and unstable)
				DHS	40			
Mohan and Kumar (2019) [36]	Retrospective cohort	India	6 Months	PFN	30	45	18	Mixed (stable and unstable)
				DHS	24	60	16	
Nargesh et al. (2013) [37]	RCT	India	30 Months	PFN	48	68	15	Mixed (stable and unstable)
				DHS	48	67	11	
Parker et al. (2012) [38]	RCT	United Kingdom	12 Months	PFN	300	82.4	52	Mixed (stable and unstable)
				DHS	300	81.4	69	
Patel et al. (2021) [39]	Prospective cohort	India	12 Months	PFN	25	NR	NR	Mixed (stable and unstable)
				DHS	25			
Patel et al. (2025) [40]	Prospective cohort	India	18 Months	PFN	40	72.1	NR	Mixed (stable and unstable)
				DHS	40	71.5		
Pehlivanoglu et al. (2021) [41]	Retrospective cohort	Turkey	6 Months	PFN	57	74.7	21	Stable
				DHS	65	72	31	
Sevinç et al. (2020) [42]	Prospective cohort	Turkey	12 Months	PFN	56	78.9	27	Mixed (stable and unstable)
				DHS	66	77.1	39	
Sharma et al. (2018) [14]	RCT	India	6 Months	PFN	31	60.67	19	Stable
				DHS	29	62.27	19	
Singh et al. (2019) [43]	RCT	India	6 Months	PFN	30	72.76	9	Mixed (stable and unstable)
				DHS	30	69.33	16	
Thusoo et al. (2024) [44]	Retrospective cohort	India	12 Months	PFN	60	57.16	20	Mixed (stable and unstable)
				DHS	140	59.35	60	
Wang et al. (2019) [45]	RCT	China	NR	PFN	57	NR	NR	Mixed (stable and unstable)
				DHS	57			
Xu et al. (2010) [46]	RCT	China	12 Months	PFN	51	78.5	15	Unstable
				DHS	55	77.9	16	
Yadav et al. (2016) [47]	Prospective cohort	India	6 Months	PFN	38	55.64	26	Mixed (stable and unstable)
				DHS	54	55.81	22	
Yeganeh et al. (2016) [48]	Prospective cohort	Iran	6 Months	PFN	75	66.68	NR	Unstable
				DHS	65	63.5		
Yu et al. (2016) [49]	Retrospective cohort	China	48 Months	PFN	110	72.02	51	Stable
				DHS	112	73.05	57	
Zehir et al. (2015) [50]	RCT	Turkey	15.95 Months	PFN	96	77.22	37	Unstable
				DHS	102	76.86	39	
Zeng et al. (2017) [51]	Retrospective cohort	China	12 Months	PFN	110	74.34	40	Stable
				DHS	112	75.16	45	

**Table 3:** Quality assessment of included studies using Newcastle-Ottawa Scale

Study ID	Selection	Comparison	Assessment	Overall
Anshul et al. (2022) [20]	4	1	2	Good
Canbeyli et al. (2021) [21]	4	1	3	Good
Cho and Lee (2016) [24]	4	1	2	Good
Chua et al. (2013) [25]	4	1	2	Good
Duan et al. (2017) [27]	4	1	2	Good
Duymus et al. (2019) [28]	3	2	3	Good
Garg et al. (2022) [29]	4	2	3	Good
Guo et al. (2017) [31]	3	1	2	Fair
Jonnes et al. (2016) [33]	4	2	2	Good
Mohan and Kumar (2019) [36]	3	1	2	Fair
Patel et al. (2021) [39]	3	1	3	Good
Patel et al. (2025) [40]	4	2	3	Good
Pehlivanoglu et al. (2021) [41]	3	2	2	Good
Sevinç et al. (2020) [42]	3	1	2	Fair
Thusoo et al. (2024) [44]	4	2	3	Good
Yadav et al. (2016) [47]	3	2	2	Good
Yeganeh et al. (2016) [48]	4	1	2	Good
Yu et al. (2016) [49]	4	2	3	Good
Zeng et al. (2017) [51]	4	2	3	Good

Mean Operative Time (in Minutes)

**Figure 2:**
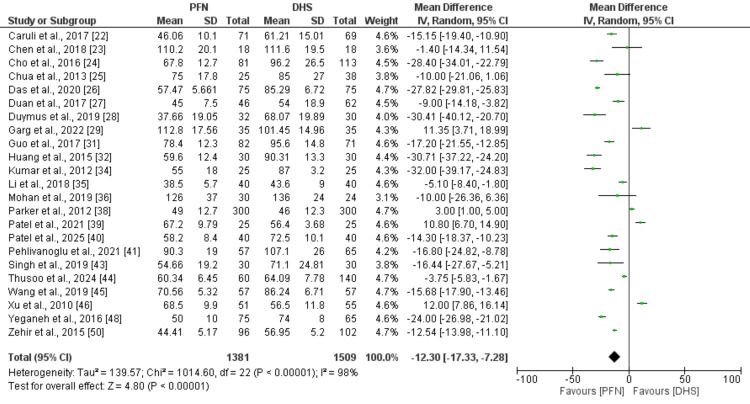
Comparison of mean operative time between two groups Reference study: [22-29,31-32,34-36,38-41,43-46,48,50] PFN: proximal femoral nail; DHS: dynamic hip screw

Figure 2 presents the pooled analysis comparing the mean operative time between PFN and DHS. The pooled analysis showed that the mean operative time was significantly lower in PFN compared to DHS (MD: -12.30, 95% CI: -17.33 to -7.28). High heterogeneity was reported among the study results (I² = 98%).

Intraoperative Blood Loss

**Figure 3:**
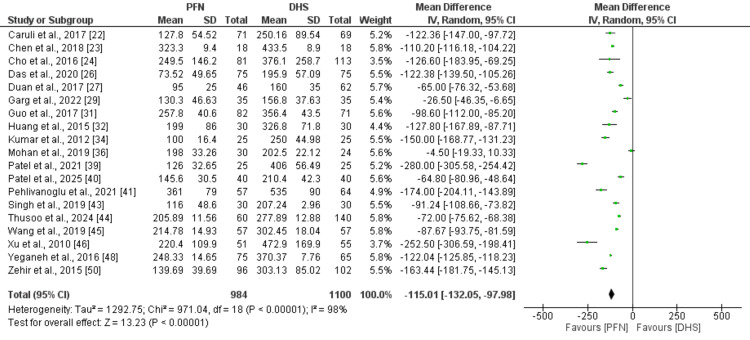
Comparison of intra-operative blood loss between two groups Reference study: [22-24,26-27,29,31-32,34,36,39-41,43-46,48,50] PFN: proximal femoral nail; DHS: dynamic hip screw

Figure 3 presents the pooled analysis comparing intraoperative blood loss between PFN and DHS. The pooled analysis showed that mean intraoperative blood loss was significantly lower in the PFN group compared to the DHS group (MD: -115.01, 95% CI: -132.05 to -97.98). High heterogeneity was reported among the study results (I² = 98%).

Secondary Outcomes

In this meta-analysis comparing PFN and DHS for the management of trochanteric fractures, functional outcomes and recovery parameters showed some differences between the two fixation methods, and the results are shown in Table 4. The Harris Hip Score was slightly higher in the PFN group compared with DHS (MD: 2.89; 95% CI: -0.69 to 6.47), although the difference was not statistically significant, and heterogeneity was high (I² = 99%). Patients treated with PFN achieved full weight-bearing significantly earlier than those treated with DHS (MD: -2.21 days; 95% CI: -3.32 to -1.10; I² = 96%), suggesting a potential advantage of PFN in early mobilization. Similarly, the Mobility Score favored PFN, with a small but non-significant improvement over DHS (MD: 0.38; 95% CI: -0.05 to 0.81; I² = 70%). Overall, these findings suggest that PFN may facilitate earlier postoperative recovery and weight-bearing compared with DHS, although both techniques provide comparable functional outcomes in the longer term.

**Table 4:** Analysis of secondary outcomes

Outcomes	Mean Difference (95% CI)	I^2^
Harris Hip Score	2.89 (-0.69 to 6.47)	99%
Full Weight Bearing (in Days)	-2.21 (-3.32 to -1.10)	96%
Mobility Score	0.38 (-0.05 to 0.81)	70%

Safety Outcomes

In terms of postoperative complications, the pooled analysis demonstrated that PFN was associated with a significantly lower risk of total complications compared with DHS (RR: 0.46; 95% CI: 0.31-0.68; I² = 44%), as shown in Table 5. Similarly, the risk of infection was significantly reduced in the PFN group (RR: 0.37; 95% CI: 0.21-0.62; I² = 0%). In contrast, there was no significant difference between PFN and DHS with respect to implant failure (RR: 1.02; 95% CI: 0.48-2.17; I² = 27%) or mortality (RR: 0.95; 95% CI: 0.58-1.53; I² = 0%). These findings suggest that PFN may reduce overall complication and infection rates compared with DHS, while the two fixation methods show comparable outcomes regarding implant failure and death.

**Table 5:** Safety outcomes

Outcomes	RR (95% CI)	I^2^
Total Complications	0.46 (0.31 to 0.68)	44%
Infection	0.37 (0.21 to 0.62)	0%
Implant Failure	1.02 (0.48 to 2.17)	27%
Death	0.95 (0.58 to 1.53)	0%

Discussion

This comprehensive meta-analysis of 33 studies provides robust evidence comparing PFN and DHS fixation in patients with intertrochanteric femur fractures. Our findings demonstrate several clinically significant advantages favoring PFN, particularly in operative parameters and early recovery outcomes, while maintaining comparable long-term functional results and mortality rates.

The present analysis revealed that PFN fixation requires significantly less operative time and results in substantially lower intraoperative blood loss compared to DHS. These findings align with previous meta-analyses by Backman et al. and Cai et al., who reported similar advantages for intramedullary fixation [52-53]. The reduced operative time with PFN can be attributed to its minimally invasive approach, requiring smaller incisions and less soft tissue dissection, compared to the extensive lateral approach necessary for DHS placement [11]. In elderly patients, who often present with multiple comorbidities and limited physiological reserves, shorter anesthesia exposure and reduced blood loss represent clinically meaningful benefits that may translate to decreased perioperative morbidity and faster recovery [54].

The substantial reduction in blood loss observed with PFN is particularly relevant, given that elderly patients are at increased risk for anemia-related complications and may have limited cardiac reserve to compensate for acute blood loss. Another study similarly demonstrated that intramedullary fixation techniques preserve the fracture hematoma and minimize surgical trauma, contributing to an enhanced bone healing environment [55]. However, the high heterogeneity (I² = 98%) observed in both operative parameters suggests significant variation in surgical techniques, implant designs, and institutional factors across studies, warranting careful interpretation of these findings.

Our analysis demonstrated that PFN-treated patients achieved full weight-bearing significantly earlier than DHS patients, which represents a clinically important advantage in elderly populations, where prolonged immobilization increases risks of pneumonia, deep vein thrombosis, and muscle deconditioning [56]. Early mobilization has been consistently associated with improved functional recovery and reduced mortality in elderly hip fracture patients [57]. The biomechanical advantages of intramedullary fixation, including superior load-sharing characteristics and proximity to the mechanical axis of the femur, likely contribute to this earlier weight-bearing capability [58].

A particularly noteworthy finding was the significantly lower total complication rate and infection risk associated with PFN fixation. The reduced infection rate can be attributed to the minimally invasive nature of intramedullary fixation, which preserves the soft tissue envelope and reduces wound complications [10]. The comparable implant failure rates between PFN and DHS suggest that modern cephalomedullary nails have overcome historical concerns about cut-out and mechanical complications, when appropriate surgical technique is employed. Recent advances in implant design, including helical blade technology and anti-rotation features, have significantly improved the mechanical performance of intramedullary devices [12]. However, implant selection should still consider fracture stability, with unstable patterns (AO/OTA 31-A2 and 31-A3) potentially benefiting more from intramedullary fixation due to superior biomechanical properties [59].

The comparable mortality rates between PFN and DHS groups align with previous systematic reviews and reflect the multifactorial nature of mortality in elderly hip fracture patients [6]. Factors such as pre-fracture functional status, comorbidity burden, and time to surgery often have a greater impact on survival than implant choice. This finding reinforces that both fixation methods are viable options, when selected appropriately based on fracture characteristics and patient factors.

The high heterogeneity observed across most outcomes reflects significant variability in study populations, fracture classifications, surgical techniques, and follow-up protocols. Future research should focus on standardizing outcome measures and stratifying results based on fracture stability patterns. Additionally, the predominance of studies from developing regions may limit generalizability to healthcare systems with different resources and protocols.

Future research should prioritize large-scale, multicenter RCTs with standardized outcome measures and longer follow-up periods to better assess functional outcomes and quality of life. Studies should stratify results based on fracture stability patterns (AO/OTA classification) to determine optimal implant selection for specific fracture types. Cost-effectiveness analyses comparing PFN and DHS across different healthcare settings are needed to guide resource allocation decisions. Additionally, investigation of newer implant designs, including helical blade technology and anti-rotation features, warrants comparative evaluation. Research should also focus on identifying patient-specific factors and fracture characteristics that predict superior outcomes with each fixation method, to enable personalized treatment approaches.

## Conclusions

This meta-analysis of 33 studies provides compelling evidence supporting the clinical advantages of PFN over DHS fixation for intertrochanteric femur fractures. PFN demonstrated superior perioperative outcomes, including significantly reduced operative time and intraoperative blood loss, which are particularly beneficial for elderly patients with limited physiological reserves. The earlier achievement of full weight-bearing with PFN facilitates improved mobilization and potentially reduces immobilization-related complications. Importantly, PFN was associated with significantly lower total complication and infection rates, while maintaining comparable implant failure and mortality outcomes. Although high heterogeneity across studies limits definitive conclusions, the consistent pattern favoring PFN across multiple outcome measures suggests its preferential use in appropriate clinical scenarios. However, implant selection should remain individualized based on fracture stability patterns, patient comorbidities, and surgeon expertise, to optimize clinical outcomes.
